# Effect of Piezoresistive Behavior on Electron Emission from Individual Silicon Carbide Nanowire

**DOI:** 10.3390/nano9070981

**Published:** 2019-07-06

**Authors:** Peng Zhao, Yu Zhang, Shuai Tang, Runze Zhan, Juncong She, Jun Chen, Ningsheng Xu, Shaozhi Deng

**Affiliations:** State Key Laboratory Optoelectronic Materials and Technologies, Guangdong Province Key Laboratory of Display Material and Technology, and School of Electronics and Information Technology, Sun Yat-sen University, Guangzhou 510275, China

**Keywords:** silicon carbide nanowire, piezoresistive effect, electronic transport, in situ electric measurement, pulse-voltage driving, electron emission

## Abstract

The excellent properties of silicon carbide (SiC) make it widely applied in high-voltage, high-power, and high-temperature electronic devices. SiC nanowires combine the excellent physical properties of SiC material and the advantages of nanoscale structures, thus attracting significant attention from researchers. Herein, the electron vacuum tunneling emission characteristics of an individual SiC nanowire affected by the piezoresistive effect are investigated using in situ electric measurement in a scanning electron microscope (SEM) chamber. The results demonstrate that the piezoresistive effect caused by the electrostatic force has a significant impact on the electronic transport properties of the nanowire, and the excellent electron emission characteristics can be achieved in the pulse voltage driving mode, including lower turn-on voltage and higher maximum current. Furthermore, a physical model about the piezoresistive effect of SiC nanowire is proposed to explain the transformation of electronic transport under the action of electrostatic force in DC voltage and pulsed voltage driving modes. The findings can provide a way to obtain excellent electron emission characteristics from SiC nanowires.

## 1. Introduction

Wide-bandgap semiconductor materials are one of the leading contenders for powered electronic devices, light-emitting diodes, transducers, and high-electron-mobility transistors. Silicon carbide (SiC) is known as a significant wide-bandgap semiconductor material owing to its high thermal conductivity, low thermal expansion coefficient, and good chemical stability. It is also considered as one of the promising materials potentially used in various microelectromechanical systems (MEMS) sensors owing to its large Young’s modulus [1,2,3,4,5,6]. Compared to the bulk counterparts, one-dimensional (1D) SiC nanomaterials possess even more unique morphology features and outstanding electrical–mechanical properties because of their low dimensionality, quantum confinement, and shape effect, which also make them very promising for use in electron emitters [7,8,9,10,11,12], sensitive sensors [13,14,15,16,17,18], and so on. Compared with other 1D nanostructure emitters [19,20], SiC nanowires have low turn-on fields, low threshold fields, and good current emission stabilities for its high field enhancement factor, low electron affinity, excellent mechanical properties, and high chemical stability [21,22,23].

For the applications of SiC nanowires in electronic devices, the electronic transport properties of SiC nanowires play a decisive role in the performance of electronic devices. Owing to the poor conductivity caused by the large bandgap in intrinsic SiC nanowires, their application in high-power and high-current electronic devices is limited, and hence their excellent performance is not fully utilized. To address these limitations, the improved electronic transport properties of SiC nanowires have been achieved via surface modification [24,25,26] and by controlling doping concentrations and doping element [27,28,29]. Metallic conductivity has been achieved by heavy doping [30] and the effect of nanometer size [9]. In addition, a piezoresistive effect was exhibited in SiC nanowires [16], which will become an important factor affecting their electronic transport characteristics under the working status of external stresses. Thus, it is necessary to study the relationship between the piezoresistive effect and electronic transport properties in the case of electrostatic stretching caused by external electric field.

In this paper, the electron vacuum tunneling emission characteristics of an individual SiC nanowire affected by piezoresistive effect are investigated using the in situ electric measurement in a scanning electron microscope (SEM) chamber. The results demonstrate that the piezoresistive effect caused by the electrostatic force has a significant impact on the electronic transport properties of the nanowire, and excellent electron emission characteristics can be achieved in the pulse-voltage driving mode, including lower turn-on voltage and higher maximum current. Furthermore, a physical model about the piezoresistive effect of the SiC nanowire is proposed to explain the transformation of electronic transport under the action of electrostatic force in DC-voltage and pulsed voltage driving modes. The findings can provide a way to obtain excellent electron emission characteristics from SiC nanowires.

## 2. Materials and Methods

### 2.1. Preparation of Individual SiC Nanowires on W Needle

The SiC nanowires were taken from the Sinet Advanced Materials Co. Ltd. (Changsha, China). SiC Nanowires, they were produced through a modified chemical vapor deposition process in which they were grown at 1800 °C using chloroform and silane as the source. The diameter of SiC nanowires was distributed within the range of 100 nm to 600 nm. To obtain thinner nanowires, the SiC nanowires were oxidized by oxygen using a tube furnace at 950 °C. Next, hydrofluoric acid etching was adopted to remove the layer of oxidation to obtain the pure surface of SiC nanowires. After that, an individual SiC nanowire was placed and soldered on the top of a tungsten needle using the electron beam-induced carbon deposition technique.

### 2.2. Morphology and Structure Characterization of SiC Nanowire

The morphology of an individual SiC nanowire was characterized using a scanning electron microscope (SEM, Zeiss Supra 55, Carl Zeiss AG, Oberkochen, Germany). The SiC nanowire was also examined using a transmission electron microscope (TEM, FEI Titan3 G2 60-300, FEI, Hillsboro, OR, USA) operated at 300 kV, and by performing Raman spectroscopy using an excitation laser of 532 nm at room temperature in the back-scattering configuration (Renishaw Invia, Renishaw, Wotton-under-Edge, Gloucestershire, UK).

### 2.3. In Situ SEM Electrical Measurement

The in situ measurement of an individual SiC nanowire was carried out in a SEM vacuum chamber with a pressure of 1 × 10^−4^ Pa. The in situ measurement instruments consisted of 4 piezoelectric ceramic manipulators, and the minimum step of each manipulator—with a maximum breakdown voltage of 500 V—was 10 nm. The in situ electrical tests were carried out using two tungsten needles-controlled manipulators, in which a SiC nanowire was placed and soldered to two different tungsten needles using the electron beam-induced carbon deposition technique. The electron vacuum tunneling emission properties of the individual SiC nanowires were characterized separately to demonstrate its performance under the DC and pulse driving modes. A tungsten needle, melted in a hemispherical shape on the top using high current, was set at 1 μm above the top surface of the SiC nanowire to apply the anode voltage. The current–voltage (I-U) characteristics driven by DC voltage were recorded using a Keithley 6487 Picoammeter. The I-U characteristics, which were driven by the pulsed voltage of frequency 200 Hz and 50% duty ratio, were collected by reading the voltage drop of the series load resistance connected with cathode using a high-precision digital oscilloscope (Tektronix MDO3024, Tektronix, Beaverton, OR, USA).

### 2.4. Simulation Method

The drawing force of tungsten needle is simulated by finite element method. The simulation includes three parts. Firstly, the physics of the solid mechanics is chosen and geometric structures are built according to the corresponding SEM images (the geometric parameters of tungsten needle and SiC nanowire are obtained from the SEM images). Then, input the mechanical parameter of each material and set the boundary conditions (boundary load is used to simulate the drawing force). Finally, mesh the geometric structure properly and calculate the result.

## 3. Results and Discussion

### 3.1. Property Analysis and In situ Electric Measurement Scheme for SiC Nanowire

**Figure 1:**
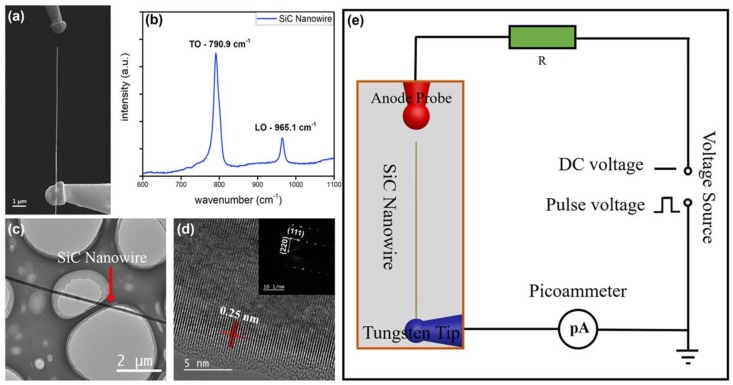
(**a**) SEM image of an individual SiC nanowire emitter and tungsten needle anode probe. (**b**) Raman spectra of SiC nanowire. (**c**) Low-magnification TEM image of SiC nanowires. (**d**) High-resolution TEM image of SiC nanowire and inset—corresponding selected area electron diffraction (SAED) image of SiC nanowire. (**e**) Schematic of in situ electron vacuum tunneling emission measurement.

A typical individual SiC nanowire electron emitter is shown in Figure 1a. The SiC nanowire is ~72 nm in diameter and ~10.4 μm in length and has a better alignment. Figure 1b shows the Raman spectra of SiC nanowire. It can be observed from the low-magnification TEM micrograph (Figure 1c) that the sample is a nanowire rather than a tapered structure. Figure 1d shows the high-resolution transmission electron microscopy (HRTEM) image and the selected area electron diffraction (SAED) image of the SiC nanowire. The above results indicate that it is a monocrystalline SiC nanowire. The nanowire has the atomic spacing of ~0.25 nm and the growth direction is [111]. The energy dispersive x-ray (EDX) analysis shows that the nanowires are pure and contain only Si and C (as shown in Figure S1). Figure 1e shows the schematic of the in situ electron vacuum tunneling emission measurements. The individual SiC nanowire was placed and soldered on the top of a tungsten needle using the electron beam-induced carbon deposition technique, and another tungsten needle was set at 1 μm above the top surface of the SiC nanowire as an anode to apply the voltage.

### 3.2. Piezoresistive Effect of Individual SiC Nanowire

**Figure 2:**
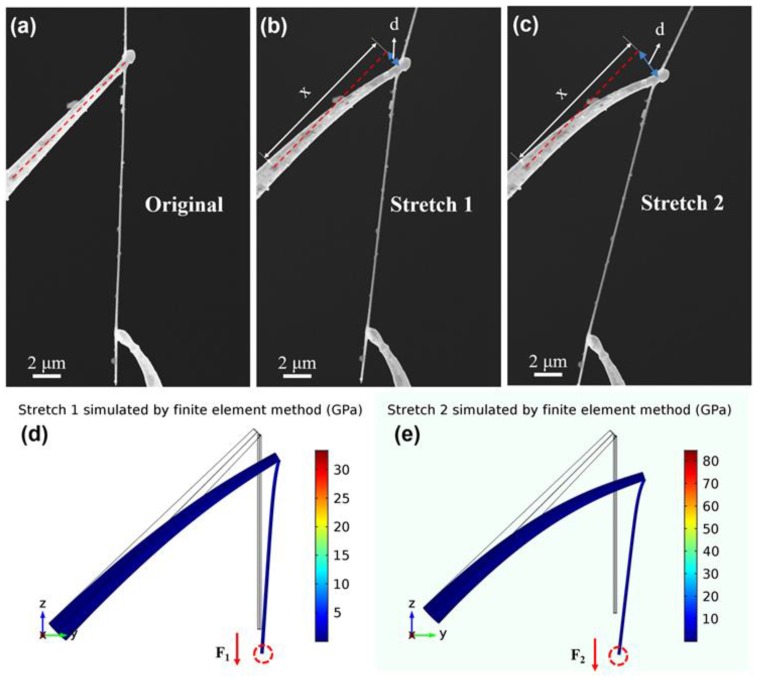
(**a**) SEM image of SiC nanowire in the original state, in which the SiC nanowire was not stretched. (**b**) SEM image of SiC nanowire in low stretching state. (**c**) SEM image of SiC nanowire in high stretching state. (**d**) Simulation result of stretch 1 state according to figure (b). (**e**) Simulation result of stretch 2 state according to figure (c).

To obtain the piezoresistive properties of the SiC nanowire in our experiments, the in situ electric measurement was performed in a SEM chamber with two piezoelectric ceramic manipulators. First, an individual SiC nanowire was fixed on the top of a tungsten needle, which was controlled by a manipulator. Another tungsten needle with a vimineous tip was in firm contact with the other side of the SiC nanowire, forming an angle of approximately 50 degrees with the nanowire, as shown in Figure 2a. The SiC nanowire is in an original state and is not stretched. In this state, the electrical properties of the SiC nanowire were characterized by in situ electrical measurements. A voltage ranging from −200 V to +200 V was applied to the SiC nanowire through two tungsten needles to obtain the I-U curves. It is the original resistance (*R*_0_) of the SiC nanowire. Next, the SiC nanowire was pulled by drawing force by moving the tungsten needle in the lower area of Figure 2a backwards through a manipulator until the tungsten needle with vimineous tip began to bend. As shown in Figure 2b, the tungsten needle tip is bending under the action of drawing force from SiC nanowire, this state is named stretch 1. Similarly, the I-U curve in the stretch 1 state was obtained by in situ electrical measurements. Third, the manipulator was moved continuously, and the tungsten needle tip was pulled by a larger drawing force, as shown in Figure 2c. This state is named stretch 2, and the I-U curve in the stretch 2 state was also obtained by in situ electrical measurements. To ensure the elastic deformation range, the morphology of tungsten needle and the resistance of the SiC nanowire were also characterized after removing the drawing force. When the drawing force from the SiC nanowire was withdrawn, the tungsten needle with vimineous tip returned to its initial state and the resistance of the SiC nanowire could be restored to the initial state (as shown in Figure S2).

Under the action of the drawing force of SiC nanowire, the vimineous tip from the tungsten needle began to bend, and the bending degree increases with an increase in drawing forces. To explore the relationship between the bending distance *d* and the drawing force *F*, the finite element method was used to simulate the drawing force. As mentioned by the literature, the Young’s modulus of tungsten nanowires is 332 GPa [31], and the Young’s modulus of SiC nanowires is 550 GPa [32]. A boundary load was used to apply drawing force at the end of SiC nanowire, marked by the red dashed circle, with direction along the negative z-axis, as shown in Figure 2d,e. In the simulation, the bending distance *d* increased with the increase of the applied drawing force *F*. As indicated in Figure 2b,c, the bending distance *d*_1_ was 1.4 μm in the state of stretch 1, and the bending distance *d*_2_ was 2.3 μm in the state of stretch 2. According to the simulation results shown in Figure 2d,e, the corresponding drawing forces *F_1_* and *F_2_* were calculated to be about 15 μN and 26 μN, respectively.

**Figure 3:**
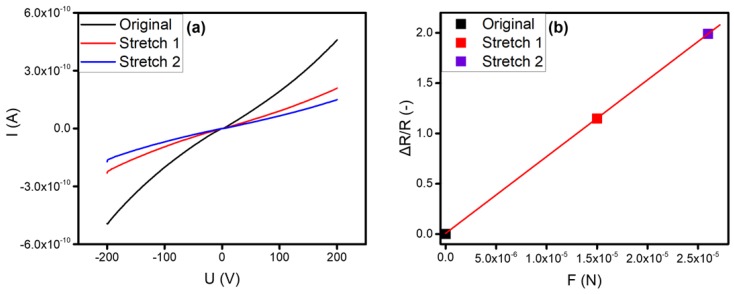
(**a**) Current versus voltage (I-U curves) of SiC nanowire for conductivity in different stretching states. (**b**) The relationship between relative resistance change (∆*R/R*) of SiC nanowire and tensile force (*F*).

As indicated in Figure 3a, I-U curves on the above three states were carried out for exhibiting the obvious piezoresistive effect of SiC nanowire, and the resistance of the SiC nanowire increased with the increase of drawing forces. As the W-SiC contact is Schottky, all I-U curves in Figure 3a exhibit a similar nonlinearity. To calculate the resistances of SiC nanowire, the equation *R =* ∆*V/*∆*I* was used, where ∆*V* is the voltage variations between two measured points in the linear sections under a positive bias (>150 V) of the I-U curves in Figure 3a, and ∆*I* is the corresponding current change, respectively. Thus, the relative resistance change (Δ*R*/*R*_0_) was calculated using I-U curves, as shown in Figure 3a. In addition, the relationship between the relative resistance change and the applied drawing force (*F*_1_, *F*_2_) was also plotted, as shown in Figure 3b. The relative resistance changes linearly increased with the drawing force, that is, the resistance of SiC nanowire changes significantly when a large tensile force is applied.

### 3.3. Electron Emission Behavior of an Individual SiC Nanowire

**Figure 4:**
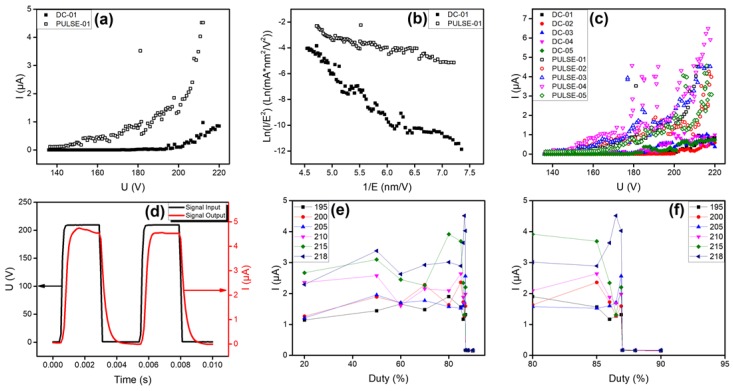
(**a**) Comparison of electron vacuum tunneling emission properties of SiC nanowire in DC and pulsed driving modes and (**b**) corresponding FN plots. (**c**) Diagram to illustrate the electron emission properties of 5 samples in different driving modes. (**d**) Pulsed waveform under pulsed driving mode collected by high-precision digital oscilloscope. (**e**) Diagram of current versus pulsed duty ratio under different pulsed peak voltages and (**f**) focus on the area of the image where the current drops sharply.

The electron vacuum tunneling emission of an individual SiC nanowire driven by DC and pulsed mode was characterized. The surface and electronic transport properties of the emitter had a distinct influence on the electron emission current. Prior to recording any emission current versus voltage (I-U curves), the SiC nanowire was subjected to a pretreatment to desorb the adsorbed gas molecule on the surface until the emission current tended to stabilize. The electron emission characteristics of the individual SiC nanowire under different driving modes are shown in Figure 4. According to FN curves (Figure 4b), the plots exhibit a linear behavior, confirming that the electron emission from an individual SiC nanowire follows the vacuum tunneling emission. As shown in Figure 4a, the SiC nanowire driven by pulsed voltage exhibits a better emission performance. The turn-on voltage of the SiC nanowire in pulsed driving mode is as low as 148 V, whereas the turn-on voltage in DC driving mode is 203 V (the turn-on voltage is defined as the voltage at which the emission current is 0.2 μA). The maximum current of the SiC nanowire driven by DC voltage is only 1.0 μA, correspondingly, the current density is 2.5 × 10^4^ A/cm^2^. By contrast, the pulsed driving mode allowed the SiC nanowire to obtain a larger emission current at almost the same voltage. The maximum current reached was 4.5 μA, correspondingly, the current density was 1.1 × 10^5^ A/cm^2^. Compared with individual carbon nanotubes [33,34], although SiC nanowire has a high turn-on voltage in DC driving mode, the maximum emission current of individual SiC nanowires can still reach several microamps or more in the pulse drive mode. It can be seen that the field emission performance of SiC nanowires is comparable to that of carbon nanotubes. It demonstrates that SiC nanowires have great application potential in field emission devices.

Table 1 and Figure 4c present a comparison of field emission properties of 5 typical samples in DC and pulsed driving modes. The results indicate that the pulsed driving mode has a much higher emission current than the DC driving mode for the SiC nanowire, both in the region of low voltage and high voltage. It is worth mentioning that the pulsed driving mode in the region of high emission current density can reduce heat effect to avoid the breakdown of emitters [35]. However, as reported by S. L. Chen, et al. [36], heat effect is conducive to the field emission ascribed to the increase of the electron–hole pairs. This is contrary to our results. To understand this opinion, the relationship between emission current and duty ratio in pulsed driving mode was studied, the electron emission of the SiC nanowire was carried out at 195 V, 200 V, 205 V, 210 V, 215 V, and 218 V. Figure 4d shows a typical pulse waveform with 200 Hz and 50% duty ratio. As shown in Figure 4e,f, the emission current changed briefly in the region with duty ratio less than 85%, and the emission current decreases rapidly with the increase in duty ratio when the duty ratio is more than about 85%. The emission current of pulsed driving mode was closer to the DC driving mode when duty ratio was close to 87%. Thus, the above results demonstrate that the electron emission properties of the SiC nanowire under pulsed driving mode were affected by electronic transport properties besides the field enhancement factor *β* and work function *ϕ*.

### 3.4. Theoretical Analysis of Electronic Transport Behavior for an Individual SiC Nanowire

The behaviors of nanowires affected by electrostatic force have been reported, such as electrostatic deflections [37,38], electromechanical resonances [39,40], electromechanical self-oscillations [41,42], structural damage [43,44], and field evaporation [45]. Thus, the effect of changing stress under electrostatic force on the electronic transport characteristics of SiC nanowire is a matter of concern.

Referring to the emitter model in Figure 1a,e, the electrons in SiC nanowire aggregate towards the top due to the applied anode voltage. According to the floating sphere model [46], as an approximation, the charge *Q* of SiC nanowire equal to the following expression:

(1) $$Q=-4\pi \varepsilon_{0}rhE,$$

where *ε*_0_ is vacuum dielectric constant, *r* is the radius of SiC nanowire, *h* is the height of emitter, and *E* is the macroscopic electric field. *E* is defined by

(2) E=U/d,

where *d* is vacuum gap between the emitter and the anode electrode, and *U* is the voltage applied across the vacuum gap. An electrostatic force *F* acting on the tip of SiC nanowire, where electrons accumulate, can be expressed as

(3) $$F=QE=-4\pi \varepsilon_{0}rhE\cdot E=-4\pi \varepsilon_{0}rh\frac{U^{2}}{d^{2}}.$$

In the case of Figure 1a, the height *h* of the emitter—which is roughly defined as the length of SiC nanowire—radius *r* of SiC nanowire, applied voltage *U*, and vacuum gap *d* is 10.4 μm, 36 nm, 218 V, and 1 μm, respectively. The calculated results show that the electrostatic force acting on the tip of SiC nanowire is 1.98 μN, which is the same order of magnitude as the drawing force inducing a significant piezoresistive effect. This means that the electronic transport properties of SiC nanowire were changed significantly by the stretching of electrostatic force during the electron emission process. In other words, under DC driving condition, a large resistance is generated in the SiC nanowire because of the continuous stretching of the electrostatic force, which causes the electron transport to be impeded. Thereby, the increase of the emission current is limited in DC driving mode. Contrarily, in pulsed driving mode, SiC nanowire is not stretched by electrostatic force during low-level period. The electrons at the top of the SiC nanowire can be effectively supplemented by the built-in electric field in this period. Therefore, a larger emission current can be obtained. When the low-level period is too short, the electrons could not be effectively replenished, and the emission current of the SiC nanowires also drops rapidly. This is consistent with the results of Figure 4e.

**Figure 5:**
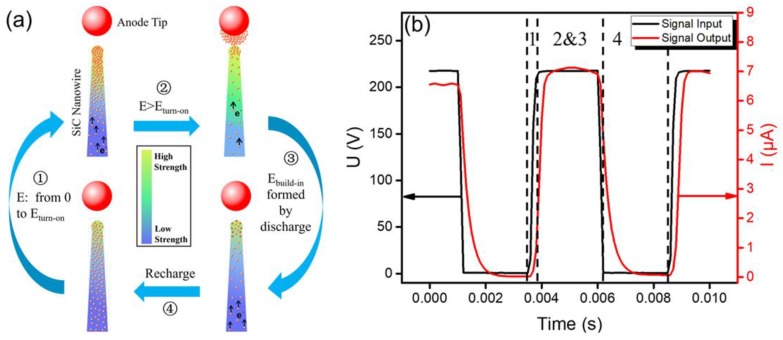
(**a**) Schematic illustration of electron emission and pumping-like electron recharge process of SiC nanowire in pulsed driving mode. (**b**) The schematic U&I-Time picture showing the corresponding cycle processes related to those indicated in (a).

To illustrate the above opinion, a pumping-like electron recharge process was used to explain the electron emission behavior of SiC nanowire in the pulsed driving mode, whose corresponding model is shown in Figure 5a.

The electron emission of SiC nanowire in the pulsed driving mode is supposed to consist of four processes. First, the electrons propelled by rising voltage accumulated from the root to the top in SiC nanowire, indicated by ① in Figure 5a. In this process, the resistance of SiC nanowire increased with rising voltage due to the piezoresistive effect generated by stretching of the electrostatic force, which impedes the supply of electrons. The next two processes can be considered to occur simultaneously, as indicated by ② and ③ in Figure 5b. In process ②, electrons emit from the top of SiC nanowire when the applied field is larger than the turn-on electric field. Meanwhile, because a large number of electrons escaped from the surface into the vacuum, and electrons supplied from the root were limited, a mass of holes was generated within the top of SiC nanowire, thereby forming a built-in electric field inside the nanowire. This process is indicated as ③ in Figure 5a. Further, when the applied field was close to 0 V/μm, the piezoresistive effect generated by stretching of the electrostatic force disappeared, hence, reducing the resistance of the SiC nanowire. So, the electrons were replenished easily and rapidly from the root to the top of SiC nanowire by means of built-in electric field. This process is indicated as ④ in Figure 5a. The above process repeats itself so that SiC nanowire in the pulsed driving mode can continue to achieve larger emission currents. The piezoresistive effect of SiC nanowire caused by electrostatic force was stable in an equilibrium state, and it should be independent of the pulse measurement. Furthermore, while the duty ratio exceeded the threshold value, the field emission current in the pulsed driving mode was close to the current in the DC mode because the electron supplement was not sufficient.

## 4. Conclusions

In summary, the electron vacuum tunneling emission characteristics of an individual SiC nanowire affected by the piezoresistive effect are revealed. The electron emitter of the individual SiC nanowire is placed and soldered using the electron beam-induced carbon deposition technique, its morphology features and structural properties are investigated using SEM, TEM, and Raman spectroscopy. The SiC nanowire exhibits considerable piezoresistance in mechanical tensile force. The electron vacuum tunneling emission behaviors of the individual SiC nanowire driven by DC and pulsed modes are characterized, and results show that the piezoresistive effect has a significant influence on the electronic transport properties due to the stretching of electrostatic forces. The theoretical model of a pumping-like electron recharge process has been developed to explain the results. The supply of electrons is limited in the DC driving mode, and for the electron emission driven by the pulsed voltage, the sufficient recharge time gives the possibility to achieve higher emission current from SiC nanowire. This study provides a solution to achieve high current electrical characteristics of SiC nanowires in the presence of electrostatic forces.

## Figures and Tables

**Table 1:** Comparison of the electron emission properties of 5 typical samples.

Samples	Maximum Current Driven by DC Voltage [μA]	Maximum Current Driven by Pulsed Voltage [μA]	Increment [μA]	Percentage of Increment
1	0.97	4.52	3.55	366%
2	0.62	4.27	3.65	588%
3	1.03	4.53	3.50	339%
4	1.07	6.49	5.42	506%
5	0.74	4.56	3.82	516%

## Supplementary Materials

The following are available online at https://www.mdpi.com/2079-4991/9/7/981/s1, Figure S1: (**a**) A representative EDX spectrum of SiC nanowire (**b**) A typical element mapping of C within the SiC nanowire. (**c**) A typical element mapping of Si within the SiC nanowire. (**d**) A typical high angle annular dark field imaging (HAADF) of SiC nanowire by scanning transmission electron microscope (STEM), especially, the red circle area was test position of EDX spectrum shown in figure (**a**). Figure S2: (**a**) SEM image of SiC nanowire in the original state (**b**) SEM image of SiC nanowire in recovery state after removing drawing force. (**c**) Current versus voltage (I-U curves) of SiC nanowire for conductivity in original states, stretch 2 state, and recovery state.
