# Supplementary material for: Effect of Piezoresistive Behavior on Electron Emission from Individual Silicon Carbide Nanowire
Source: Nanomaterials (Basel). 2019 Jul 6;9(7):981. doi: 10.3390/nano9070981 (PMC6669601; doi:10.3390/nano9070981)
Supplement: Supplementary file 1 [file nanomaterials-09-00981-s001.pdf]

Supplementary Materials

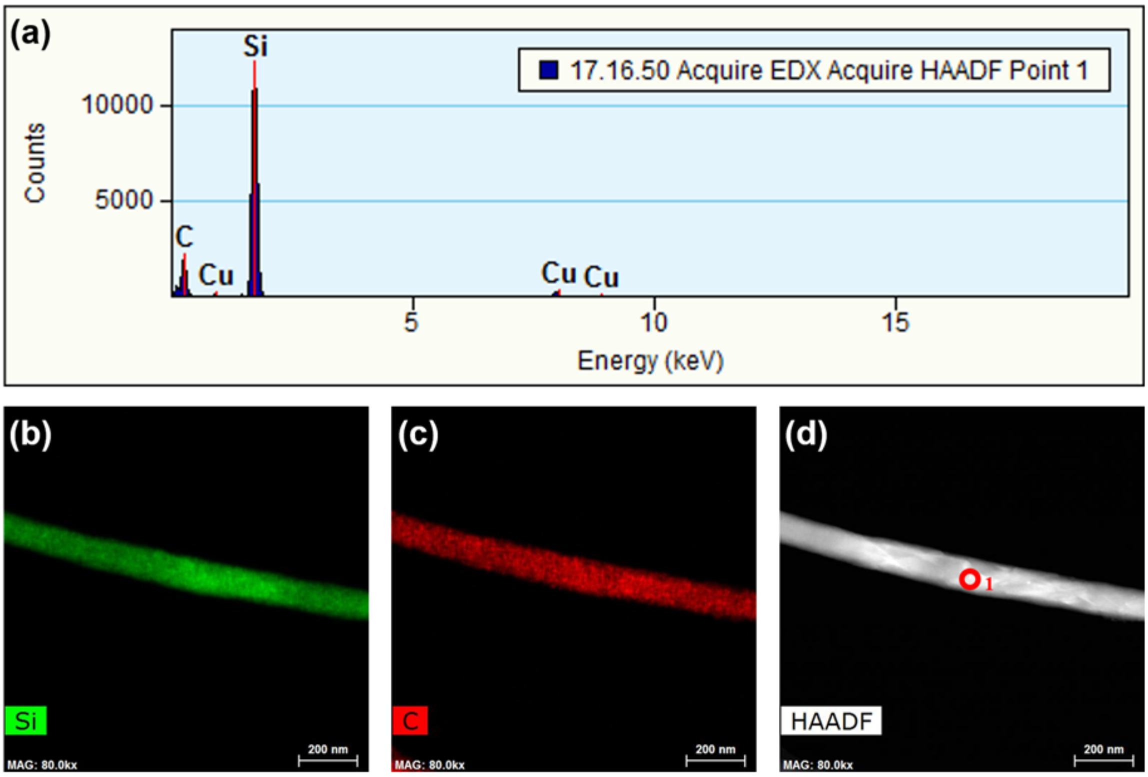

Figure S1. (**a**) A representative EDX spectrum of SiC nanowire (**b**) A typical element mapping of C within the SiC nanowire. (**c**) A typical element mapping of Si within the SiC nanowire. (**d**) A typical high angle annular dark field imaging (HAADF) of SiC nanowire by scanning transmission electron microscope (STEM), especially, the red circle area was test position of EDX spectrum shown in figure (**a**).

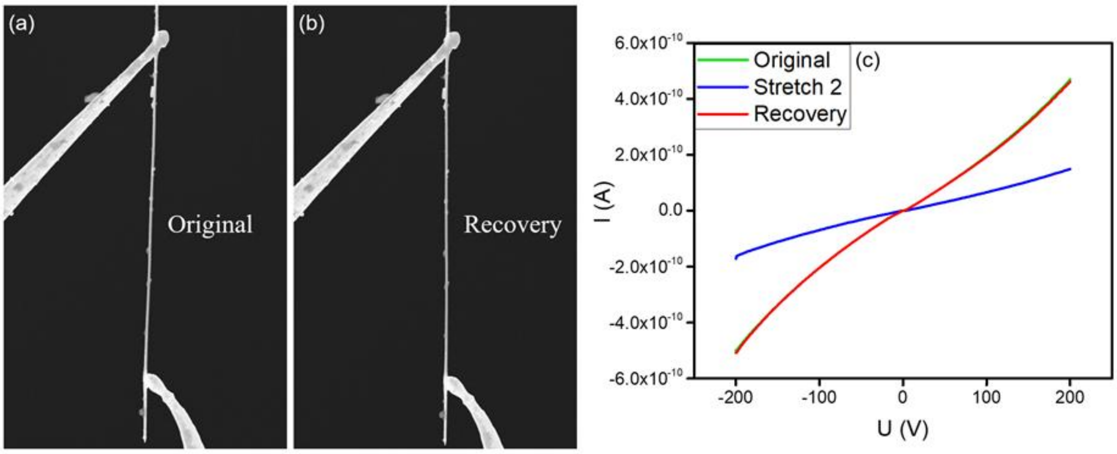

Figure S2. (**a**) SEM image of SiC nanowire in the original state (**b**) SEM image of SiC nanowire in recovery state after removing drawing force. (**c**) Current versus voltage (I-U curves) of SiC nanowire for conductivity in original states, stretch 2 state, and recovery state.
